# The transition zone: an essential functional compartment of cilia

**DOI:** 10.1186/2046-2530-1-10

**Published:** 2012-07-02

**Authors:** Katarzyna Szymanska, Colin A Johnson

**Affiliations:** 1 Section of Ophthalmology and Neurosciences, Leeds Institute of Molecular Medicine, St. James’s University Hospital, Leeds, UK

**Keywords:** Cilia, Transition zone, IFT, Ciliopathies

## Abstract

Recent studies of the primary cilium have begun to provide further insights into ciliary ultrastructure, with an emerging picture of complex compartmentalization and molecular components that combine in functional modules. Many proteins that are mutated in ciliopathies are localized to the transition zone, a compartment of the proximal region of the cilium. The loss of these components can disrupt ciliary functions such as the control of protein entry and exit from the cilium, the possible trafficking of essential ciliary components, and the regulation of signaling cascades and control of the cell cycle. The discovery of functional modules within the primary cilium may help in understanding the variable phenotypes and pleiotropy in ciliopathies.

## Review

Cilia are microtubule-based, hair-like organelles that occur on the apical surface of most mammalian cells in G_0_/G_1_ of the cell cycle [1], with the exception of bone marrow-derived cells [2]. Defects in cilia structure or function are the cause of a suite of congenital conditions known as ciliopathies, which now include polycystic kidney disease, nephronophthisis, Senior-Løken syndrome, Bardet-Biedl syndrome (BBS), Joubert syndrome (JBTS) and Meckel-Gruber syndrome (MKS). Most of these conditions vary in the severity of the clinical phenotype, and display extensive allelism [3] and pleiotropy [3-7].

## Ultrastructure of the cilium

**Figure 1:**
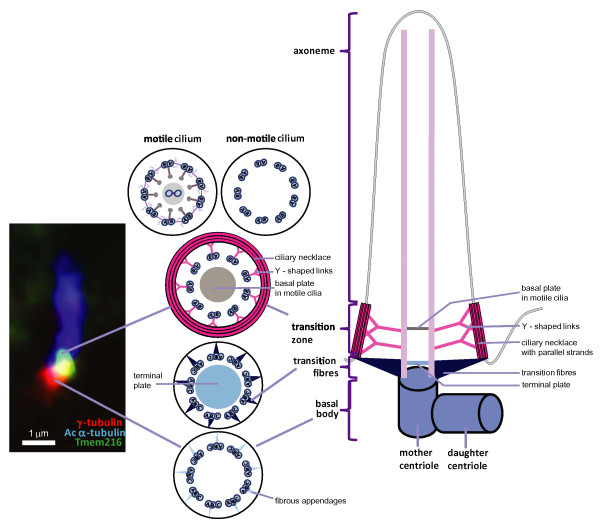
**This figure shows schematic representation of cilia ultrastructure with cross-sections at the level of the basal body, transition fibers, transition zone and axoneme in motile and non-motile cilia.** On the left hand side, is an enlarged immunofluorescence micrograph of a single primary cilium on an IMCD3 epithelial cell immunostained for the cilia marker acetylated α-tubulin (blue), the basal body marker γ-tubulin (red), and transmembrane protein (TMEM) 216 (green) which localizes to the transition zone. Scale bar = 1 μm.

There are three general types of cilia, differentiated on the basis of their microtubule structure (Figure 1). Cilia with the canonical “9 + 2”microtubule pattern are motile: Motile cilia occur on epithelial cells in the lungs, in the olfactory bulb, in the inner ear as a kinocilium, and in the reproductive tracts of both sexes. Motile cilia in the respiratory tract perform “whip”-like movements to mediate fluid flow [8], although olfactory cilia, despite their 9 + 2 microtubule pattern, are thought to be immotile [9] and kinocilia are moved by deflection [10]. During embryonic development nodal cilia play a crucial role in left-right patterning. They are located at the embryonic node, and although they have a “9 + 0” microtubule pattern they mediate a leftward flow at the node in a “whirlpool”-like manner due to the retention of dynein arms between microtubules. The leftward flow is thought to transport morphogens that are essential for the first step of symmetry breaking in the developmental of the mammalian body plan [2]. The third type of cilia is an immotile (“9 + 0”) primary cilium, which is now known to participate in diverse roles in cell signaling, chemosensation, mechanosensation and thermosensation [11].

In quiescent cells, cilia are derived from a mother centriole that migrates to the apical surface of the cell and matures into the basal body. The mother centriole consists of a barrel-shaped structure of nine triplets of microtubules, each triplet built from A-, B- and C-tubules (Figure 1). The basal body consists of both mother and daughter centrioles, but only the former acts as a matrix for the subsequent nucleation of microtubules during the formation of a cilium. The mother centriole can also be distinguished from the daughter by the presence of fibrous distal and subdistal appendages [12]. During ciliogenesis, the doublets containing A- and B-tubules are extended to form the ciliary axoneme by a process of intraflagellar transport (IFT) [13] and the centriolar appendages mature into transition fibers [14]. Once the basal body is docked, the axoneme begins to be assembled. The microtubules mediate anterograde transport towards the tip of cilia by kinesin motors carrying IFT-complex B proteins and other cargo proteins. In turn, cytoplasmic dyneins mediate retrograde movement of IFT-complex A towards the proximal regions of the cilium. IFT transport plays a crucial role in cilia assembly and disassembly as well as in transporting signaling components. Dyneins are also components of more general motor protein complexes responsible for minus-end, microtubule-based motile processes. IFT mediates both assembly and resorbtion of the cilium, and processing of key intermediates of signaling cascades [15].

## The transition zone

The most proximal region of a cilium is called the “ciliary gate” and can be divided into two structurally distinct sub-regions: the transition fibers and the transition zone (Figure 1) [16]. For many years, the ciliary gate was observed in TEM (transmission electron microscopy) cross-sections [16-19] but its function remained unknown. These early observations suggest that the ciliary gate forms at the very early stages of ciliogenesis that precede IFT. The basal body terminates with the end of the C-tubule and the beginning of the transition fibers. More distally, the axoneme of the cilium is then established. In motile cilia, the boundary between the axoneme and the transition zone is known as the basal plate and is thought to take part in the nucleation of the central microtubules (Figure 1) [20]. The appearance of the transition zone varies between species and cell-type [21], but the basic structural components appear to be conserved. For example, the so-called “connecting cilium” in photoreceptor cells of mammals is a ciliary transition zone that extends between the outer and inner segment of photoreceptors [22], and its extended structure was a factor in enabling the biochemical purification of outer segments and the identification of the photoreceptor sensory cilium proteome [23].

Transition fibers (Figure 1) emerge from the B-tubules of the basal body triplet microtubules just before the end of the C-tubule and form a “pinwheel-like” structure on TEM cross-sections. The tips of transition fibers are thought to anchor microtubules to the plasma membrane, although the composition of transition fibers is still largely unknown. In some species and in motile cilia the terminal plate (often visualized as an electron-dense aggregate on TEM) is observed in this area. No function has been ascribed for the terminal plate, although in *Tetrahymena* it was shown to be enriched in centrin [24]. Transition fibers are observed on the mature mother centriole and they may play a role in anchoring to the plasma membrane through CEP164 [25] and ODF2 (outer dense fibre 2)/cenexin [26]. In *Chlamydomonas*, IFT52 was observed on transition fibers as well [27], suggesting that they have a role in docking the IFT and motor proteins required for ciliogenesis. Although the complete protein composition of those fibers is still unknown, it is thought that they take part in creating a pore complex, similar to nuclear pores, which is required for transporting proteins in and out of cilia [28].

Moving up toward the ciliary tip and on the other side of the ciliary gate, the so-called “Y-shaped” linkers and the ciliary necklace are observed (Figure 1), the latter a characteristic structure of the transition zone. Y-shaped linkers are structures connecting the outer doublets of microtubules to the plasma membrane and the ciliary necklace [29]. Their detailed protein structure is as yet uncharacterized and their shape and name is species specific [20,29-34]. The ciliary necklace is a specialized structure that consists of several parallel strands of intramembrane particles and their number is species and cell-specific. The identity of these strands is unknown, but they encircle the ciliary membrane spacing from the plasma/ciliary membrane boundary to the basal plate [20]. Y-shaped linkers and the ciliary necklace are especially visible in the elongated transition zone structure of connecting cilia in photoreceptors [35], and the latter has been shown to contain CEP290 in *Chlamydomonas*[36] and RPGRIP1L in *C.elegans*[19]. In motile cilia, the ciliary necklace coincides with the minus ends of the central pair of microtubules at the basal plate [20]. This region is enriched in γ-tubulin [37], which suggests that this is the location for nucleation and stabilisation of the central pair of microtubules. Similar to the transition fibers, the transition zone has been proposed to regu-late ciliary protein composition in *Chlamydomonas*, *C. elegans* and mammalian cells [19,36,38] by regulating intracellular trafficking to and from the cilium. However, the molecular details of protein sorting at the transition zone remain to be discovered.

## Trafficking to cilia

It is difficult to define a simple model for the mechanisms of protein targeting to cilia, with several competing models described below and reviewed elsewhere [39-41]. Although the identity of ciliary cargo proteins is becoming clearer, the mechanisms of their intracellular transport are still mysterious. One model assumes the existence of characteristic protein recognition sequences that mediate correct targeting. A focus of research has been the ciliary transport of transmembrane proteins, which has required the identification of specific protein sequences necessary for targeting and the identification of modules that recognize these sequences [42-44]. These include, for example, the N-terminal RVxP sequence for the PKD2 protein [44], and the C- terminal VxPx sequence for rhodopsin [43].

A second model involves vesicle trafficking and exocytosis, although the direct vesicle transport of membrane proteins through the ciliary gate is unlikely because the vesicle size exceeds 60 nm. Instead, proteins are thought to be transported in vesicles from the Golgi apparatus to a specific docking site at the periciliary base. The exocyst complex is then thought to tether the vesicles, presumably directing the fusion of vesicles with the ciliary membrane mediated by SNAREs (soluble *N*- ethylmaleimide sensitive factor receptors) and the Rab family of small GTPases [45]. SNAREs are present at the surface of the exocyst complex to allow proteins to pass the ciliary diffusion barrier and to cross to the ciliary membrane [45], implying an active transport process [41]. This model of trafficking would therefore be analogous to that proposed for nuclear pores where importins and exportins are utilized. IFT proteins may mediate this transport machinery since they are enriched at the level of transition fibers [27]. Once trafficked through the ciliary gate, proteins may then be incorporated as cargo proteins in the IFT complexes and transported along the ciliary axoneme. The third model suggests that IFT proteins create clusters in the trans-Golgi network that coat vesicles and ensure targeted trafficking to the cilium [46]. The clusters would later become IFT complexes still attached to cargo proteins.

However, studies of Rab8 seem to confirm the vesicle theory, and clearly suggest that Rab GTPases coordinate with each other in the regulation of vesicular trafficking during primary ciliogenesis. Rab8 is a member of the Rab family of small GTPases that regulates membrane traffic from endosomal compartments to the cell surface [47] as well as mediating transport, docking and fusion of vesicles with acceptor modules at the base of the primary cilium [48]. Rab8 plays an essential role in ciliogenesis [49-51] and acts downstream of Rab11 and Rabin8, the guanine nucleotide exchange factor (GEF) that activates Rab8 [52]. An additional downstream binding partner of Rab8 is CEP290, which has been shown to localize to the pericentrosomal compartment, basal body and transition zone [53,54]. CEP290 plays an important role at the ciliary gate, where together with other proteins containing C2/B9-related domains predicted to bind phospholipids [55], it is proposed to be involved in membrane/vesicle trafficking and fusion [53]. In particular, CC2D2A, a C2 domain-containing protein, is now also suggested to mediate vesicle trafficking and fusion since it localizes to the photoreceptor connecting cilium/transition zone, and appears to facilitate protein transport through Rab8-dependent processes [56].

Another possible mechanism of protein targeting to cilia is through the BBSome complex. This is proposed to mediate trafficking of transmembrane proteins to the ciliary membrane and consists of seven highly conserved proteins, one novel protein and a coat-like structure [50]. The BBSome directly recognizes cilia targeting sequences and is the major effector of Arl6/BBS3 (an Arf-like GTPase). Rabin8, the GEF that activates Rab8, has also been shown to interact with the BBSome [50]. The BBSome is not directly required for cilia formation in most tissues [57] but its failure to deliver important receptors and transmembrane proteins to the cilium is thought to result in cell signaling failure and organ-specific pathological abnormalities. Proteins, after fulfilling their function in the cilium, have to be removed from it. This function is suggested to be also mediated by the BBSome. In *Chlamydomonas* it was observed that the BBSome and IFT particles co-localize, and the BBSome may play a docking function for the retrograde IFT complex A [58]. Proteins targeted to be removed from cilia may also be ubiquitinated, then accumulated at the base of cilia for targeted degradation by the endocytic pathway [59].

## Recent advances in characterizing the transition zone

**Figure 2:**
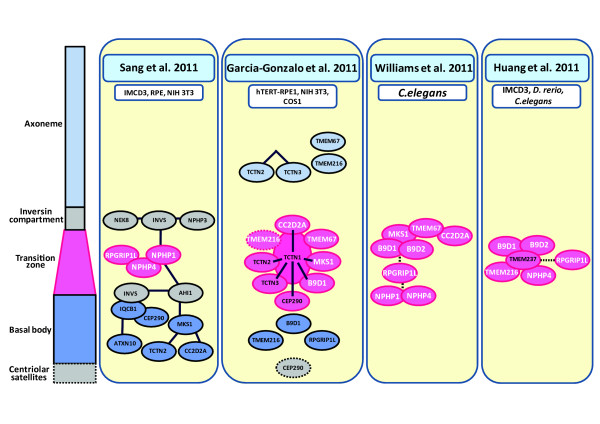
**Localization of the indicated proteins (coloured ovals) to sub-ciliary compartments (pale blue, axoneme; grey, inversin compartment; pink, transition zone; dark blue, basal body; grey with dashed square , centriolar satellites) are represented based on biochemical and genetic interaction data from four recent studies of the composition of the transition zone (Sang *et al. *2011,Garcia-Gonzalo *et al. *2011,Williams *et al. *2011 and Huang *et al. *2011; each indicated at the top of each section).** White text indicates common components identified by different studies; dark lines indicate an interaction identified by MS/MS; a dashed line indicates a genetic interaction; overlapping ellipses indicated a direct interaction. The model system(s) used to generate these data are also indicated for each study.

In five recent papers, the composition of protein complexes at the ciliary gate and transition zone was investigated. In two of the studies [54,60], the authors used the G-LAP tandem-affinity method, a stringent proteomic strategy for the identification of interacting proteins. It allowed the authors not only to identify three interconnected functional modules, but to also identify three new ciliopathy genes: Sang et al. 2011 identified *ATXN10* and *TCTN2*, and Garcia-Gonzalo et al. 2011 identified *TCTN1*. The first module, the “nephronophthisis NPHP module” identified in Sang et al. 2011, was composed of NPHP1/nephrocystin-1, which was previously suggested to take part in the regulation of cargo and IFT ciliary entry [61]. NPHP4 and RPGRIP1L (also components of the NPHP module) were also localized to the transition zone and cell-cell boundaries. *In vitro* studies showed that this module was not essential for ciliogenesis, but it may play an important part in epithelial morphogenesis and the establishment of tissue architecture. On the other hand, a second module (the “JBTS module”) that contains IQCB1 and CEP290 is essential for cilia formation. Proteins in the third module (the “MKS module” containing MKS1, CC2D2A and TCTN2) are involved in neural tube development and Sonic Hedgehog signaling. The authors proposed the existence of an additional ciliary segment located distal to the transition zone in the proximal part of cilia, which they called the “inversin compartment” (Figure 2). The function of this section of the cilium remains to be understood, but several proteins were found to be localized in it, notably NEK8, INVS/inversin and NPHP3 [62]. INVS and AHI1/jouberin were found to play a bridging role between these three modules. There were no interactions observed between the NPHP-JBTS-MKS modules and the BBS proteins, and no overlap with IFT complex proteins [60].

In Garcia-Gonzalo et al. 2011, the authors identified mutations in *TCTN1* as a cause of Joubert syndrome. They showed that TCTN1/tectonic-1 interacts with TCTN2, TCTN3, MKS1, B9D1 and CC2D2A and, under some circumstances, with CEP290, TMEM67/meckelin and TMEM216. They showed that TCTN1 localizes to the transition zone and that it is required for transition zone localization of MKS1 and TMEM67. During detailed studies they observed other proteins at the transition zone: TCTN2, TCTN3 and TMEM67 (all three of which also localized elsewhere in the cilium); MKS1 and CEP290 (also localized to centriolar appendages); CC2D2A and B9D1 (also localized to the basal body) and NPHP4. RPGRIP1L was localized to the basal body like TMEM216, which was also observed at distal regions of the cilium and occasionally at the transition zone (Figure 1). The authors stated that the Tectonic module (containing TCTN1, TCTN2 and TCTN3) and MKS proteins localize to the transition zone where they regulate ciliogenesis and ciliary membrane composition in a tissue-specific manner, and dysfunction of the transition zone is a basic defect causing human ciliopathies [54].

Williams and colleagues, 2011 conducted their research on *C. elegans* which has ciliated sensory neurons in amphid sensory cells. They grouped the investigated proteins into two modules: MKS/MKSR (containing MKS1, B9D1, B9D2, TMEM67, CC2D2A) and NPHP (containing NPHP1 and NPHP4) (Figure 2) that despite seemingly unrelated components share a common biological function. Both modules are linked through RPGRIP1L, which is responsible for endogenous localization to the transition zone for all of the other investigated proteins. Collectively, both modules are required for the early steps of ciliogenesis, namely the basal body – transition zone membrane docking and formation of the transition zone [19]. It is notable that CC2D2A has been implicated in Rab8-dependent vesicle trafficking and fusion [56], although the role of the MKS/MKSR module in mediating these processes remains unclear.

Li et al. 2011 [63] investigated TCTEX1/DYNLT, a dynein light chain subunit involved in cargo binding but that can also be recruited for functions independent of the dynein complex [64]. Active phosphorylated TCTEX1 was found to be recruited to the ciliary transition zone prior to cilia disassembly and entry into S phase of the cell cycle [63]. Furthermore, active TCTEX1 was recruited to the transition zone of neuronal progenitor cells, where it had a key role in cell cycle regulation and the probable determination of cell fate between proliferation of progenitors and differentiation into post-mitotic neurons.

In Huang *et al.* 2011, the authors identified mutations in *TMEM237/JBTS14* as a cause of Joubert syndrome-related disorders [65]. They used three model systems (*C. elegans*, zebrafish and IMCD3 cells) to investigate biochemical and genetic interactions between TMEM237 and other ciliary proteins. TMEM237 localized to the ciliary transition zone and, largely in agreement with previous studies [54,60], interacted genetically with NPHP4, TMEM216, B9D1 and B9D2. The authors also noted that TMEM237 required RPGRIP1L for localization to the transition zone in both the IMCD3 cell and *C. elegans* models, which suggests that RPGRIP1L has a structural role in mediating the scaffolding or bridging between interacting ciliary proteins [65].

Figure 2 summarizes the localization of ciliary proteins based on the results of Sang *et al.* 2011, Garcia-Gonzalo *et al.* 2011, Williams *et al.* 2011 and Huang *et al.* 2011. There are some obvious inconsistencies among the studies, which could be explained by possible differences in cilia structure between tissues and the dynamic nature of ciliogenesis and protein transport within cilia. Sang *et al.* 2011 proposed interaction modules that will be very useful in further analysis of protein-protein interactions, signaling pathways and the molecular structure of cilia. Garcia-Gonzalo *el al.* 2011 added another module containing Tectonic proteins (TCTN1, TCTN2 and TCTN3) crucial for cilia formation and the patterning of the developing embryo. Williams *et al.* 2011 described a further functional module (the “MKS/MKSR module” including MKS1, TMEM67, CC2D2A, B9D1 and B9D2). This observation confirmed the existence of a general “NPHP1-NPHP4-RPGRIP1L module” with RPGRIP1L functioning as a bridging protein. Huang *et al.* 2011 described a fourth ciliopathy gene*TMEM237* using a next-generation sequencing strategy, and confirmed the transition zone localization of RPGRIP1L, NPHP4, B9D1 and B9D2. The dynamic nature of ciliogenesis regulation by transition zone proteins has been highlighted by a fourth recent study [63].

## Conclusion

**Figure 3:**
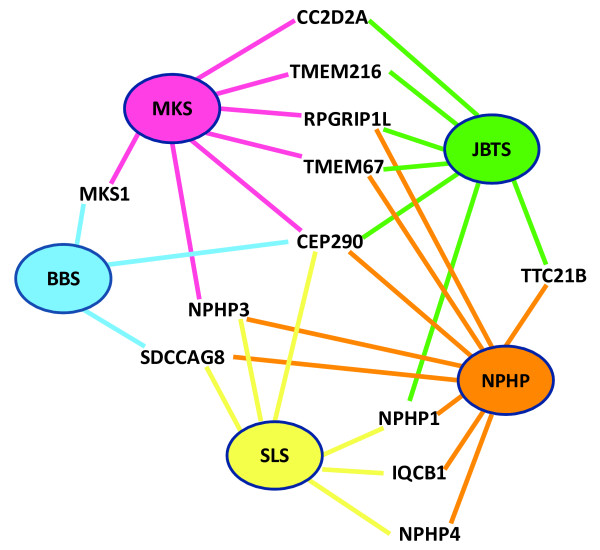
Five ciliopathies with extensive genetic heterogeneity and pleiotropy (BBS, Bardet-Biedl syndrome; JBTS, Joubert syndrome; MKS, Meckel-Gruber syndrome; NPHP, nephronophthisis; SLS, Senior-Løken syndrome) are indicated by ovals of different colour, with the ciliary proteins mutated as a known cause of each disorder indicated by the coloured lines.

In conclusion, four recent studies [19,54,60,65] support the concept of human ciliopathies being caused by sorting defects at the transition zone (Table 1), and the ciliary gate playing a crucial role in cilia assembly and selective regulation of cilia protein content. What remains unexplained is the function of ciliary modules in mediating cilia trafficking, how these could regulate the signaling cascades that are mediated by cilia, and the connection with other complexes such as the inversin module and the BBSome. It seems likely that the elucidation of these mechanistic details will begin to explain the phenotypic variability and pleiotropy of human ciliopathies (Figure 3). These could arise from either the diverse requirements of the protein composition of transition zones in different tissues, or the influence of modifier alleles in interacting components of individual functional modules such as TTC21B or RPGRIP1L [3,66]. A fifth study [63] has highlighted the complex, dynamic nature of the transition zone and a possible role of this region of the cilium in G_1_/S checkpoint control. Linking the cilium, cell cycle control and extracellular cues of signaling pathways will be a further field of intensive future work, and will no doubt bring further surprises in our understanding of the complex ultrastructure of the primary cilium.

**Table 1:** List of proteins localized to the transition zone involved in ciliopathies

**Gene**	**Localization**	**Publication**	**Ciliopathy**
*MKS1*	TZ	Garcia-Gonzalo et al, 2011; Williams et al, 2011	MKS
	BB	Sang et al, 2011	MKS
*TMEM216*	AX	Garcia-Gonzalo et al, 2011	MKS, JBTS
	TZ	Garcia-Gonzalo et al, 2011; Huang et al, 2011	MKS, JBTS
	BB	Garcia-Gonzalo et al, 2011	MKS, JBTS
*TMEM67*	AX	Garcia-Gonzalo et al, 2011	MKS, JBTS, NPHP
	TZ	Garcia-Gonzalo et al, 2011; Williams et al, 2011	MKS, JBTS, NPHP
*CEP290*	TZ	Garcia-Gonzalo et al, 2011	MKS, JBTS, NPHP, BBS, LCA, SLS
	BB	Sang et al, 2011	MKS, JBTS, NPHP, BBS, LCA, SLS
	CS	Garcia-Gonzalo et al, 2011	MKS, JBTS, NPHP, BBS, LCA, SLS
*RPGRIP1L*	TZ	Sang et al, 2011; Williams et al, 2011; Huang et al, 2011	MKS, JBTS, NPHP, LCA
	BB	Garcia-Gonzalo et al, 2011	MKS, JBTS, NPHP, LCA
*CC2D2A*	TZ	Williams et al, 2011	MKS, JBTS
	BB	Sang et al, 2011	MKS, JBTS
*TCTN2*	AX	Garcia-Gonzalo et al, 2011	MKS
	TZ	Garcia-Gonzalo et al, 2011	MKS
	BB	Sang et al, 2011	MKS
*NPHP1*	TZ	Sang et al, 2011; Williams et al, 2011	JBTS, NPHP, SLS
*NPHP4*	TZ	Sang et al, 2011; Garcia-Gonzalo et al, 2011; Williams et al, 2011; Huang et al, 2011	NPHP, SLS
*B9D1*	TZ	Garcia-Gonzalo et al, 2011; Williams et al, 2011; Huang et al, 2011	MKS
	BB	Garcia-Gonzalo et al, 2011	MKS

The proteins are described to localize to the transition zone and other ciliary compartments in recent publications. Abbreviations: *AX*, Axoneme; *BB*, Basal body; *BBS*, Bardet-Biedl syndrome; *CS*, Centriolar satellites; *JBTS*, Joubert syndrome; *LCA*, Leber congenital amaurosis; *MKS*, Meckel-Gruber syndrome; *NPHP*, nephronophthisis; *SLS*, Senior-Løken syndrome; *TZ*, transition zone.

## Abbreviations

BBS: Bardet-Biedl syndrome; IFT: Intraflagellar transport; JBTS: Joubert syndrome; LCA: Leber congenital amaurosis; MKS: Meckel-Gruber syndrome; NPHP: Nephronophthisis; TEM: Transmission electron microscopy; TMEM: Transmembrane protein; SLS: Senior-Løken syndrome; SNARE: Soluble *N*- ethylmaleimide sensitive factor receptor; GEF: Guanine nucleotide exchange factor.

## Competing interests

The authors declare no competing financial interests.

## Authors' contributions

KS and CAJ contributed equally to the preparation of the manuscript. Both authors read and approved the final manuscript.
